# Minimally-invasive approach via percutaneous femoral cannulation for the resection of intra-cardiac masses: a single center experience in the Middle-East

**DOI:** 10.1186/s13019-023-02295-1

**Published:** 2023-07-03

**Authors:** Uthman Aluthman, Mohammed A. Ashour, Salman W. Bafageeh, Abivarma Chandrakumaran, Taraji S. Alrehaili, Osama A. Abdulrahman, Ahmed F. Elmahrouk, Shalan Alaamri, Saeed A. AlGhamdi, Ahmed A. Jamjoom

**Affiliations:** 1 Cardiovascular Department, King Faisal Specialist Hospital and Research Centre, Ar Rawdah, 2865, Jeddah, 23431 Saudi Arabia; 2 College of Medicine, King Abdulaziz University, Jeddah, Saudi Arabia; 3 College of Medicine, King Saud Bin Abdulaziz University for Health Science, Jeddah, Saudi Arabia; 4 Tbilisi State Medical University, Tbilisi, Georgia; 5 College of Medicine, Umm Al-Qura University, Makkah, Saudi Arabia; 6 College of Medicine, University of Jeddah, Jeddah, Saudi Arabia; 7 King Fahad General Hospital, Jeddah, Saudi Arabia

**Keywords:** Minimally invasive surgery, Cardiac tumors, Cardiac myxoma, Angiosarcoma, Minithoracotomy, Middle East

## Abstract

**Background:**

Intra-cardiac masses are rare and challenging lesions with an overall incidence ranging of 0.02–0.2%. Minimally invasive approaches have been recently introduced for surgical resection of these lesions. Here, we evaluated our early experience using minimally invasive techniques in addressing intra-cardiac lesions.

**Methodology:**

This is a retrospective descriptive study conducted between April 2018 to December 2020. All patients were diagnosed with cardiac tumors and treated via a right mini-thoracotomy with cardiopulmonary bypass through femoral cannulation at King Faisal Specialist Hospital and Research Centre, Jeddah.

**Results:**

Myxoma was the most common pathology representing 46% of cases followed by thrombus (27%), leiomyoma (9%), lipoma (9%) and angiosarcoma (9%). All tumors were resected with negative margins. One patient was converted to open sternotomy. Tumor locations were in the right atrium, left atrium, and left ventricle in 5, 3, and 3 patients, respectively. The median ICU stay was 1.33 days. The median length of hospitalization was 5.7 days. There was no 30-days hospital mortality recorded in this cohort.

**Conclusion:**

Our early experience shows that minimally invasive resection can be performed safely and effectively for intra-cardiac masses. The minimally invasive approach using a mini-thoracotomy with percutaneous femoral cannulation can be an effective alternative in resecting intra-cardiac masses that achieves clear margin resection, quick post-operative recovery, and low rates of recurrence for benign lesions.

## Background

Intra-cardiac masses are rare and challenging lesions with an overall incidence ranging between 0.02–0.2% [1–3]. Cardiac myxoma is the most common primary benign tumor, and sarcomas are the most common malignant type [2, 3]. Even though most primary cardiac tumors are benign, they have the potential to cause severe morbidity due to systemic embolization and intracardiac obstruction [4]. Once an intra-cardiac mass is identified, it prompts surgery in order to prevent complications.

The traditional approach to the resection of these masses once diagnosed is through a median sternotomy. While very effective in achieving complete resection, there is significant surgical trauma associated with this approach. Many centres around the world have adopted a minimally invasive approach to various cardiac surgeries that have shown to achieve similar outcomes, while minimizing surgical trauma and allowing for faster postoperative recovery [5, 6]. These outcomes usually translate into higher patient satisfaction with this approach compared to the median sternotomy.

The minimally invasive approach has become especially popular among valvular surgeries, but is being adopted in other facets of cardiac surgery as well [5]. Many groups have managed resection of benign cardiac masses via this limited access approach and have reported excellent outcomes similar to conventional sternotomy [7, 8]. Despite its introduction in the late 1980s, minimally invasive cardiac surgery is still considered a rather novel approach in the Middle Eastern region with only a few centres performing them regularly. While there have been published studies utilizing this approach for resection of intra-cardiac masses from North America, Europe and East Asia, to our knowledge there have been none from the Middle Eastern region [9–11].

In this study, we evaluated our early experience of using minimally invasive techniques for the resection of intra-cardiac masses from April 2018 to December 2020 at a single institution in Saudi Arabia with 3-year follow up of these patients.

## Methods

This was a retrospective descriptive study conducted at King Faisal Specialist Hospital and Research Centre, Jeddah. We included all patients who underwent minimally invasive surgery for excision of intra-cardiac masses from April 2018 to December 2020. Patients who underwent a traditional open sternotomy procedure were excluded. The preoperative diagnosis was established in all patients via echocardiography. All patients were treated via a right mini-thoracotomy with cardiopulmonary bypass through femoral cannulation. Data were retrieved from our hospital medical records after IRB approval. Preoperative data included demographic data, clinical presentation, echocardiography, radiological data, and any other associated medical conditions. Operative data included the tumor size, origin pathology, cardiopulmonary bypass time, cross-clamp time, operative events, operative procedure, hospital course, and length of stay.

### Surgical technique

**Fig. 1:**
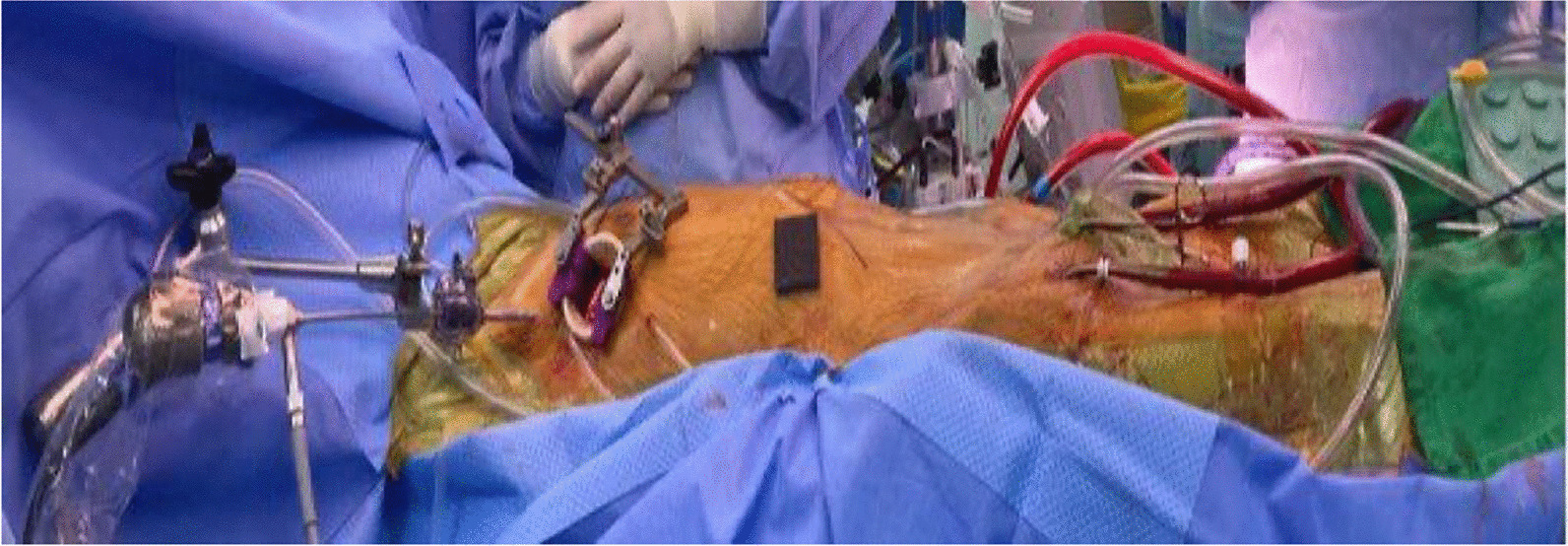
Intraoperative positioning with percutaneous cannulation of both groins

The procedure began with the induction of anaesthesia. One-lung ventilation was performed using a double lumen tube or a single tube with a bronchial blocker. Electrocardiogram (ECG), pulse oximetry, temperature monitoring, as well as arterial and central lines with/without Swan-Ganz catheters were used for routine monitoring lines. Trans-esophageal echocardiogram (TEE) probe was inserted, and defibrillator pads were applied in all cases. The patient was positioned supine and draped in a 20-degree left lateral decubitus position as seen in Fig. 1. The chest was accessed through the 4th intercostal space via a 4-cm right anterolateral mini thoracotomy incision. Percutaneous right femoral artery cannulation was performed under TEE guidance and a vascular closure device (Perclose ProGlide™, Abbott, Chicago, U.S.A) was used for closure at the end of the case. Percutaneous left femoral vein cannulation with multi-port venous cannula was performed up to superior vena cava (SVC) under TEE guidance. Cardiopulmonary bypass began after heparinization, and diaphragmatic and pericardial retention sutures were then placed. The SVC and inferior vena cava were skeletonized and then developed an intra-arterial groove. A vented cardioplegia needle was then placed in the aortic root. A transthoracic aortic cross clamp was then applied, and cold del-Nido cardioplegia was delivered antegrade to achieve diastolic arrest. A left atriotomy incision was made to expose the left atrium in cases of left atrial mass. In cases of right atrial mass, we added a SVC canula via the same mini-thoracotomy incision. Snares were applied on both cava via a 30-degree 5-mm endoscopic camera. The field was flooded with CO2, mass excised, and atria closed in routine fashion. The cross clamp was then removed, and reperfusion was initiated. Systemic heparinization was reversed with protamine. Both peripheral cannulas were removed. Finally, one right chest tube and one right Blake drain were placed. Once the haemostasias was achieved, the pericardium was closed with interrupted 4/0 Ethibond sutures (Ethicon INC, Sommerville, New Jersey). One pericostal stitch was placed to approximate the ribs and Vicrel sutures used to approximate subcutaneous tissues in running layers while monocryl was used to close the skin.

## Results

### Preoperative clinical data

Eleven patients with intra-cardiac masses underwent minimally invasive excision: seven were females (64%). The mean age was 35.67 (age range between 16 and 62). Embolic events were the main presenting symptoms (46%) followed by asymptomatic (18%), dyspnea (9%), fever of unknown origin (9%), palpitation (9%), and chest pain (9%). The four most common associated medical conditions were atrial fibrillation, embolic stroke, ischemic heart disease and systemic lupus erythematosus (19.8%) as seen in Table 1. Preoperative echocardiography showed that most of the masses were located in the atrium, five of the patients (46%) in the right, and three (27%) in the left. The remaining three (27%) were in the left ventricle. Based on echocardiography, the mass size was estimated using the length and width (cm), ranging from 10 × 12 mm up to 5.6 × 3.6 cm. Preoperative mean left ventricular ejection fraction (LVEF) was 48.6 ± 10.5% (ranging from 20 to 60%). According to the New York Heart Association (NYHA) classification, eight patients’ cardiac function was in grade I (72.7%), followed by one in each of grade II, III and IV (9%) (Table 1).

**Table 1:** Baseline patient characteristics

Variable	
Age	35.67 ± 15.29 (16–62) years
Sex (female)	64%
Pre-operative LVEF	48.6 ± 10.6% (20–60%)
*Associated medical conditions*	
Atrial fibrillation	2 (18%)
Embolic stroke	2 (18%)
Endocarditis	1 (9%)
Heart Failure	1 (9%)
Idiopathic ventricular arrhythmia	1 (9%)
Ischemic heart disease	2 (18%)
Peripheral vascular disease	1 (9%)
Rheumatic heart disease	1 (9%)
SLE	2 (18%)
TVR	1 (9%)
*Main symptoms*	
Dyspnea	1 (9%)
Embolization related	5 (46%)
Fever of unknown origin	1 (9%)
Incidentally	2 (18%)
Palpitation	1 (9%)
Chest pain	1 (9%)
*NYHA class*	
Class I	8 (72%)
Class II	1 (9%)
Class III	1 (9%)
Class IV	1 (9%)

*LVEF* left ventricular ejection fraction, *SLE* systemic lupus erythematosus, *TVR* tricuspid valve regurgitation

### Surgical outcome and pathologic findings

**Fig. 2:**
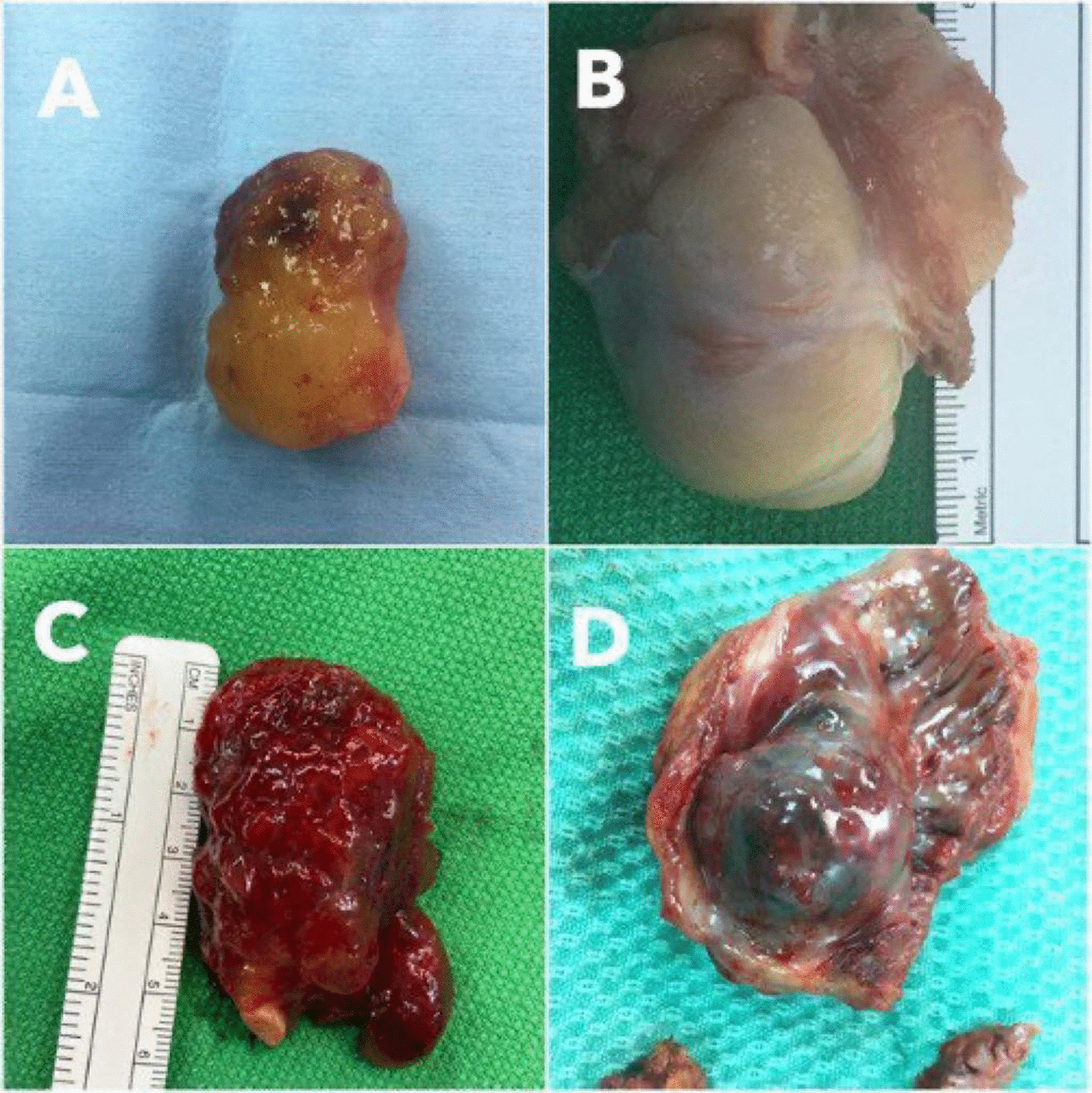
Surgical gross specimen of **A** left atrial myxoma; **B** right atrial leiomyoma; **C** left atrial myxoma; **D** right atrial angiosarcoma

**Fig. 3:**
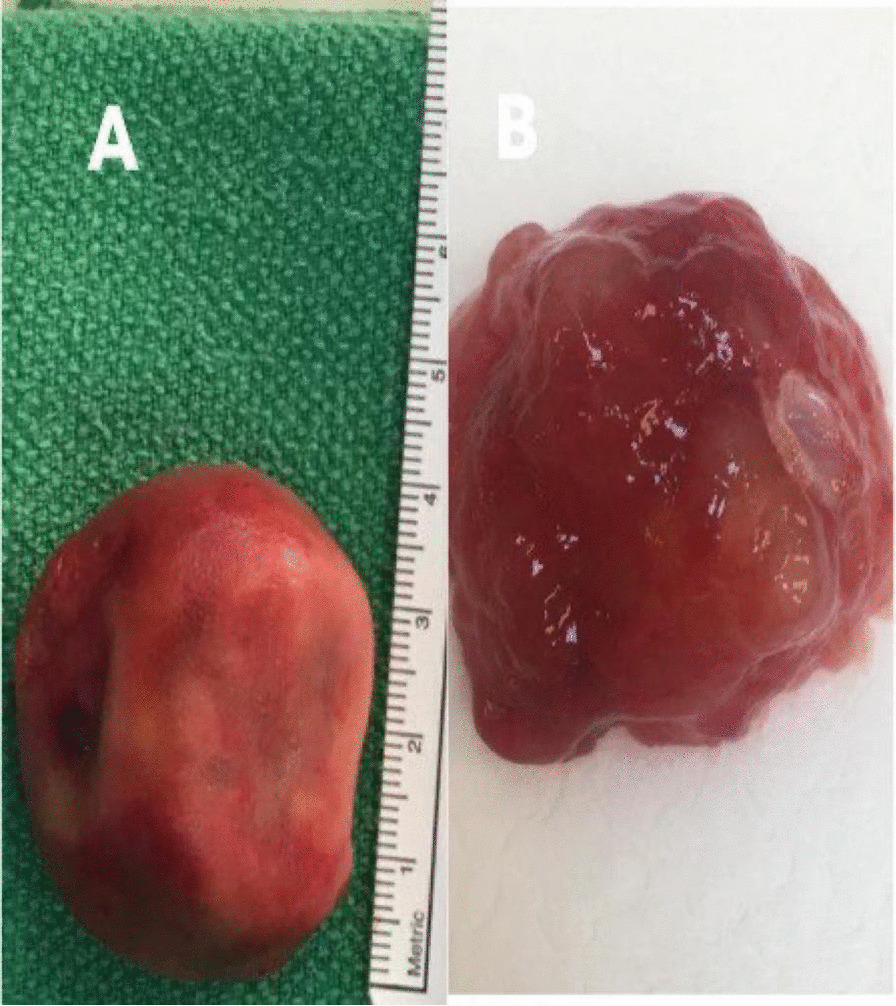
Surgical gross specimen of thrombus in **A** left atrium; **B** right atrium

The intra-cardiac mass postoperative pathology showed cardiac myxoma in five patients (46%); two in the left atrium, two in the right and one in the left ventricle (Table 2). Three patients (27%) had thrombus; one located in the left atrium, one in the right atrium and one in the left ventricle. Three had leiomyoma, lipoma and angiosarcoma (9%), which was located in the right atrium, left ventricle and right atrium respectively (Figs. 2 and 3). Only the angiosarcoma (9%) operation had to be converted to open sternotomy due to the presence of excessive adhesions and surgeon preferences. Mortality within 30 days, six months and one year were 0, 0, and 1 patient respectively. All the patients were extubated less than 12 h after surgery except for the patient with angiosarcoma. The mean ICU length of stay (LOS) was 1.33 days (ranging from 1 to 3 days). The mean hospital LOS was 5.7 days (ranging from 3 to 8 days). None of the patients had any vascular access complications, wound infection, or needed blood transfusion (Table 3).

**Table 2:** Cardiac mass characteristics

Variable	
Tumor size (L × W)	0.10 × 0.12 cm–5.6 × 3.6 cm
*Final pathological diagnosis*	
Leiomyoma	1 (9%)
Lipoma	1 (9%)
Myxoma	5 (46%)
Angiosarcoma	1 (9%)
Thrombus	3 (27%)
*Location*	
Left atrium	3 (27%)
Right atrium	5 (46%)
Left ventricle	3 (27%)

*L* Length, *W* Width

**Table 3:** Intraoperative variables and surgical outcomes

Variable	
*Intraoperative*	
Cardiopulmonary bypass time (minutes)	79.2 ± 5.1
Cross-clamp time (minutes)	42.3 ± 7.5
Conversion to sternotomy	1 (9%)
Vascular access complications	0 (0%)
Blood transfusion	0 (0%)
*Surgical outcomes*	
ICU length of stay	1.33 ± 0.70
Hospital length of stay	5.70 ± 1.49
Intubation less than 12 h	10 (91%)
Surgical site infection	0 (0%)
In-hospital mortality	0 (0%)
30-Days mortality	0 (0%)
1-year mortality	1 (9%)
2-year mortality	1 (9%)
3-year mortality	1 (9%)
New VI on follow up TEE	0 (0%)

*VI* Valvular insufficiency

### Follow-up results

The mean postoperative follow up was 23 ± 7.99 months (ranging from 12 to 36 months). It was done using echocardiogram and clinical evaluation, showing optimum recovery in the majority throughout the follow up period. There was no reoperation or recurrence of tumor. The preoperative presenting symptoms had been relieved after surgical intervention and no complications were reported. The only mortality recorded was in the angiosarcoma (9%) patient due to hepatic, spleen, lung, and bone metastasis after receiving palliative chemotherapy.

## Discussion

Minimally invasive cardiac surgery has been performed since the 1990s and has been gaining popularity in various facets like mitral-valve surgery due to reduced blood loss and pain, shorter ICU and hospital stay, and superior patient satisfaction [6, 12, 13]. Cardiac masses are generally very rare among patient populations and hence the data regarding minimally invasive resections for intra-cardiac masses are limited [2, 3]. Most studies that have reported on this approach for the resection of cardiac masses are generally from highly specialized centres in Europe, North America, and East Asia [9, 11]. Given the novelty of the approach, skilled training for surgeons, and experience with this approach there has been a dearth of studies looking at minimally invasive approaches for cardiac surgery in the Middle-East. To our knowledge this is the first study that uses the minimally invasive approach for intra-cardiac mass resection in the Middle-Eastern region.

Among our cohort of patients with a mean age of 35.6 ± 15 years, there was a higher percentage of females and the main presenting symptom was embolic stroke. Our patient population was younger compared to other published series using the minimally invasive (MI) approach [9]. Additionally, chest pain and dyspnea has been the most commonly reported presenting symptom among other published cohorts but we had a higher percentage of patients presenting with embolic stroke than chest pain or dyspnea. Regardless of cardiac tumor pathology, the main presenting symptoms in 46% of patients was embolic events such as stroke, omental infarction, pulmonary embolism, and embolism of unspecified artery. The most common site of the intra-cardiac mass in our patients was the right atrium followed by the left atrium and left ventricle. This was again in contrast to what was observed in other studies which have a higher percentage of masses in the left atrium followed then by the right atrium [9, 10]. Most of the patients in our series were in the NYHA Class I (72%), with 1 patient each in Class II (9%), III (9%) and IV (9%).

The most commonly reported intra-cardiac mass was myxoma across many studies with varying percentages of lipomas and fibroelastomas [7, 9, 10]. In our series of patients, after pathological examination the most common intra-cardiac mass was found to be myxoma (46%), thrombus (27%), leiomyoma (9%), and lipoma (9%). Because of the difficulty in assessing the mass with echocardiography, the size of the mass and associated symptoms of the patient, 3 out of 11 of our patients were found to have a thrombus. A meta-analysis of a published studies comparing the MI approach to traditional sternotomy reported a rate of thrombus among the studies ranging from 0–13% [9]. The rate observed in our cohort is especially high but we attribute this mainly to the small size of our cohort.

Due to the complexity involved in performing complete resections with clear margins through a smaller incision, one of the major concerns with the minimally invasive approach is the increased time on cardiopulmonary bypass (CPB) and cross-clamp (CC) compared to the median sternotomy approach. The mean time spent on cardiopulmonary bypass was 79.2 ± 5.1 and on cross-clamp was 42.25 ± 7.5 min. A study done in 2010 compared the outcomes of patients who underwent tumor resection either using a MI approach or traditional sternotomy. They found that there was no significant difference in the CPB times and CC times between the two groups [10]. The mean CPB time reported in their study for the MI group was 77.0 ± 4.2 min and the CC time was 41.3 ± 4.1 min, which is similar to what was seen in our cohort. However, a recent large meta-analysis that was compared the MI approach to traditional sternotomy did find a significant difference in the time spent on CPB and CC, with the MI groups having longer times [9]. Nevertheless, this did not translate into any significant difference in the clinical outcomes or postoperative complication rates. The CPB and CC times maybe prolonged with the MI approach depending on many variables such as the experience of the surgeon and complexity of the case, yet has no significant impact on the clinical outcomes.

Due to the smaller incision size associated with the MI approach, many surgeons are often concerned about the potential for inadequate resection, which may lead to a high rate of recurrence. In our cohort of 11 patients with intra-cardiac masses, 10 of them were resected with clear margins based on pathological examination. One of the patients had to be converted to median sternotomy due to extensive tumor infiltration into the pericardium and was later diagnosed with angiosarcoma on pathological examination. Our results with the MI approach for the pathologically benign lesions is similar to what was observed in other studies, who reported complete and adequate resection of the intra-cardiac masses [10, 14, 15]. The rate of recurrence for benign cardiac tumors treated with the MI approach remain low across various studies ranging from 2–3.3% after a follow up of at least 4 years [10, 15, 16]. In our patient cohort, at 3 years’ follow-up, there were no recurrences observed and no reoperations performed for patients diagnosed with the benign cardiac masses. The patients underwent regular annual follow-up, which revealed no masses on echocardiographic evaluation.

The use of the MI approach for malignant cardiac tumors remains very controversial with many recommending against this. While the use of MI approach has been published in the literature for malignant cardiac tumors, the need for greater access and complex cardiac reconstructions usually preclude the use of this approach [17]. According to some authors the MI approach may be considered for malignant lesions if it is isolated, having a diameter less than 3 cm and is expected to be completely resected [7]. Our patient who was diagnosed with angiosarcoma, initially underwent a right mini-thoracotomy similar to the other patients but was later converted to a traditional median sternotomy mainly to achieve adequate resection of the aggressive pericardial infiltration. Additionally, the patient was found to have multiple hepatic, spleen and bone metastasis on follow up. He has been treated with palliative chemotherapy but unfortunately died 11 months after the initial surgery.

The main benefits in using MICS is the reduction in postoperative recovery times, postoperative pain, ICU and hospital length of stay [6, 18]. Many studies have also reported reduction in the need for blood transfusion [18, 19]. All these factors also tend to culminate as higher rates of patient satisfaction postoperatively. In our cohort of patients, 91% needed < 12 h of intubation postoperatively with 1 patient needing 32 h. The mean LOS in the ICU was 1.33 ± 0.70 days which is similar (35.6 ± 20.6 h) to what has been reported in other series using the same approach [9, 10]. The mean LOS in hospital was 5.70 ± 1.49 days for our patients again similar to other MI series [10, 15]. Both of these variables have been reported to be significantly longer in patients who underwent resection with traditional sternotomy [8, 20, 21]. This underscores the benefit provided not only to the patient in terms of recovery times but also the ability to reduce hospital resource utilization and costs.

### Limitations

There are several limitations in our study. Due to the retrospective nature of the study it was subject to multiple biases. Although our sample represents the largest cohort of minimally invasive resection of cardiac masses in the Middle East, the absolute size is still very small. As such, our study may not have identified safety and efficacy concerns with the MI approach. However, the results shown in our cohort are similar to what has been published in other studies with the exception of the percentage of patients with thrombus. Because of the recent adoption of the minimally invasive approach by our center, it was only possible to follow up patients up to 3 years. Excluding the angiosarcoma patient, there were no recurrence or death in our group. In addition to a larger cohort, a longer follow up maybe needed to look for recurrences. All patients, however were symptom-free and had no echocardiographic evidence of recurrence on annual follow up for 3 years.

## Conclusion

Our early experience shows that minimally invasive resection can be performed safely and effectively for intra-cardiac masses. The minimally invasive approach using a mini-thoracotomy with percutaneous femoral cannulation can be an effective alternative in resecting intra-cardiac masses that achieves clear margin resection, quick post-operative recovery, and low rates of recurrence for benign lesions. By showing the feasibility and safety of this approach at our institution which recently adopted this technique, we hope more centres in the Middle East and the world will consider this approach for the resection of benign intra-cardiac masses.

## Data Availability

The data supporting the conclusions of this article is included within the article.

## Abbreviations

CC: Cross-clamp
CPB: Cardiopulmonary bypass
ECG: Electrocardiogram
LVEF: Left ventricular ejection fraction
LOS: Length of stay
MI: Minimally invasive
NYHA: New York Heart Association
SVC: Superior vena cava
TEE: Transesophageal echocardiogram
